# Optimized Intracellular Staining Reveals Heterogeneous Cytokine Production Ability of Murine and Human Hematopoietic Stem and Progenitor Cells

**DOI:** 10.3389/fimmu.2021.654094

**Published:** 2021-04-14

**Authors:** Shufeng Luo, Huiling Lin, Lan Zhu, Hai-Tian Chen, Siqian Yang, Jinheng Li, Mingyu Liu, Limin Zheng, Chong Wu

**Affiliations:** ^1^ MOE Key Laboratory of Gene Function and Regulation, School of Life Sciences, Sun Yat-Sen University, Guangzhou, China; ^2^ Collaborative Innovation Center for Cancer Medicine, State Key Laboratory of Oncology in South China, Sun Yat-Sen University Cancer Center, Guangzhou, China; ^3^ First Affiliated Hospital, Sun Yat-Sen University, Guangzhou, China

**Keywords:** hematopoietic stem and progenitor cells, cytokine, flow cytometry, infection, tumor

## Abstract

Under stress conditions, hematopoietic stem and progenitor cells (HSPCs) can translate danger signals into a plethora of cytokine signals. These cytokines, or more precisely their combination, instruct HSPCs to modify the magnitude and composition of hematopoietic output in response to the threat, but investigations into the heterogeneous cytokine expression and regulatory mechanisms are hampered by the technical difficulty of measuring cytokine levels in HSPCs at the single-cell level. Here, we optimized a flow cytometry-based method for the simultaneous assessment of multiple intracellular cytokines in HSPCs. By selecting an optimal combination of cytokine restimulation reagents, protein transport inhibitors, and culture supplements, an optimized restimulation protocol for intracellular staining was developed. Using this method, we successfully examined expression levels of granulocyte/macrophage colony-stimulating factor (GM-CSF), interleukin-6 (IL-6), and tumor necrosis factor-α (TNF-α) in murine and human HSPC subsets under steady-state or different stress conditions. Different cytokine expression patterns were observed, suggesting distinct regulatory modes of cytokine production dependent on the HSPC subset, cytokine, disease, organ, and species. Collectively, this technical advance may help to obtain a better understanding of the nature of HSPC heterogeneity on the basis of differential cytokine production.

## Introduction

Hematopoietic stem cells (HSCs) and hematopoietic progenitor cells (HPCs), residing at the top of the hematopoietic hierarchy, are responsible for the maintenance of steady-state and stress-adapted blood cell generation. In recent years, the paradigm that HSCs and HPCs (collectively referred to as HSPCs) divide in response to peripheral cytopenia has given way to one in which HSPCs can directly sense bacterial and viral components through Toll-like receptors (TLRs) and proinflammatory cytokines through cytokine receptors, thereby serving as a foundation for the immune response (1–8). These new findings indicate that HSPCs play an important role in systemic immune reactions and support the long-neglected fact that HSPCs are first responders to infection and other pathological conditions.

Proinflammatory cytokines and chemokines are critical regulators of HSPC activity (9–12). Accumulating evidence suggests that HSPCs, like their mature myeloid and lymphoid descendants, are capable of translating danger signals into a plethora of cytokines that act in an autocrine or paracrine manner to regulate stress-induced hematopoiesis (13, 14). These cytokines represent a crucial line of communication and a way to amplify signals to convey the presence of danger among the HSPC community, along with information on what cell types should be produced in response to the threat. However, this area of research remains largely unexplored, and investigations are hampered by a lack of an ideal technique for measuring the ability of HSPCs to produce cytokines. Due to the rarity and highly heterogeneous nature of HSPCs, the development of single-cell level detection methods is essential for obtaining a clear picture of HSPC cytokine production. In 2014, David Baltimore and colleagues introduced a microfluidic-based platform to quantify secreted proteins at the single-cell level. Using this technique, they found that the cytokine production ability of HSPCs exceeds that of mature myeloid and lymphoid cells not only in terms of the magnitude but also the speed and breadth (13). However, the methodological complexity, high cost, and relatively low cell throughput of this method have limited its widespread application, and single-cell, protein-level evidence in relevant studies is still mostly inferred or omitted.

Flow cytometry is one of the most commonly accepted techniques in immunology and can be easily adapted to study complex phenotypes and the type or amount of molecules produced by the cell population of interest (15, 16). Using this technique among others, we previously found that cancer is often associated with “emergency” extramedullary hematopoiesis, in which murine data suggest that cytokine-producing HSPCs may play a critical role (17, 18). However, the detection of intracellular cytokines in HSPCs, in particular simultaneously assessing multiple cytokines in human HSPCs, has been difficult. A major challenge is to identify an optimal restimulation condition that supports cells to continue producing different cytokines *in vitro* while sequestering these cytokines within the cell. Here, we optimized a flow cytometry-based method for the simultaneous assessment of multiple intracellular cytokines in human and murine HSPCs. Granulocyte/macrophage colony-stimulating factor (GM-CSF), interleukin-6 (IL-6), and tumor necrosis factor-α (TNF-α) are among the most highly induced cytokines in stressed HSPCs and play critical roles in regulating emergency hematopoiesis (9, 13, 19). With the use of this optimized protocol, we measured expression levels of these cytokines in murine and human HSPC subsets under steady-state and different stress conditions and observed different production patterns across cytokines and species.

## Materials and Methods

### Animals

All mice were maintained under specific pathogen-free conditions in the animal facilities of Sun Yat-sen University Cancer Center. C57BL/6 mice (6–8 weeks of age) were purchased from Guangdong Medical Laboratory Animal Center (Guangzhou, China). Orthotopic hepatic tumors were established as previously described (18). Briefly, 7.5 × 10^5^ Hepa1-6 tumor cells (purchased from ATCC, USA) were suspended in 25 µl of 50% basement membrane extract (Trevigen, USA) and injected into the left liver lobes of anesthetized mice. Animal experiments were performed in accordance with state guidelines and were approved by the IACUC of Sun Yat-sen University (Approval No., SYSU-IACUC-2019-B574).

### Human Blood Samples

Human peripheral blood (PB) samples were obtained from healthy donors attending the Guangzhou Blood Center (Guangzhou, China). Healthy human cord blood (CB) samples were obtained from the First Affiliated Hospital of Sun Yat-Sen University. All samples were coded anonymously in accordance with local ethical guidelines (as stipulated by the Declaration of Helsinki). The protocol was approved by the Review Board of Sun Yat-Sen University (Approval No., GZR2019-084).

### Cell Isolation and Flow Cytometry

#### Cell Isolation

For the isolation of murine HSPCs, bone marrow (BM) or spleen cells were first treated with red blood cell lysis buffer (TBD Science, China) to remove red blood cells. c-Kit^+^ cells were then sorted by magnetic-activated cell sorting (MACS) using CD117 microbeads (Miltenyi Biotec, Germany). Next, lineage (lin)^low/-^Sca1^+^c-Kit^high^ (LSK) cells were purified by flow cytometry cell sorting with a MoFlo Astrios EQ flow cytometer (Beckman Coulter, USA). To isolate human HSPCs, human PB or umbilical CB samples from healthy donors were separated by Ficoll density-gradient centrifugation. CD34^+^ cells were purified using a direct CD34 progenitor cell isolation kit, according to the manufacturer’s instructions (Miltenyi Biotec). Lin^-^CD34^+^ HSPCs were further sorted by flow cytometry cell sorting. Details on antibodies used for flow cytometry are provided in **Supplemental Table 1**.

#### Preparation of *C*ytokine-Expressing HSPCs

**Figure 1:**
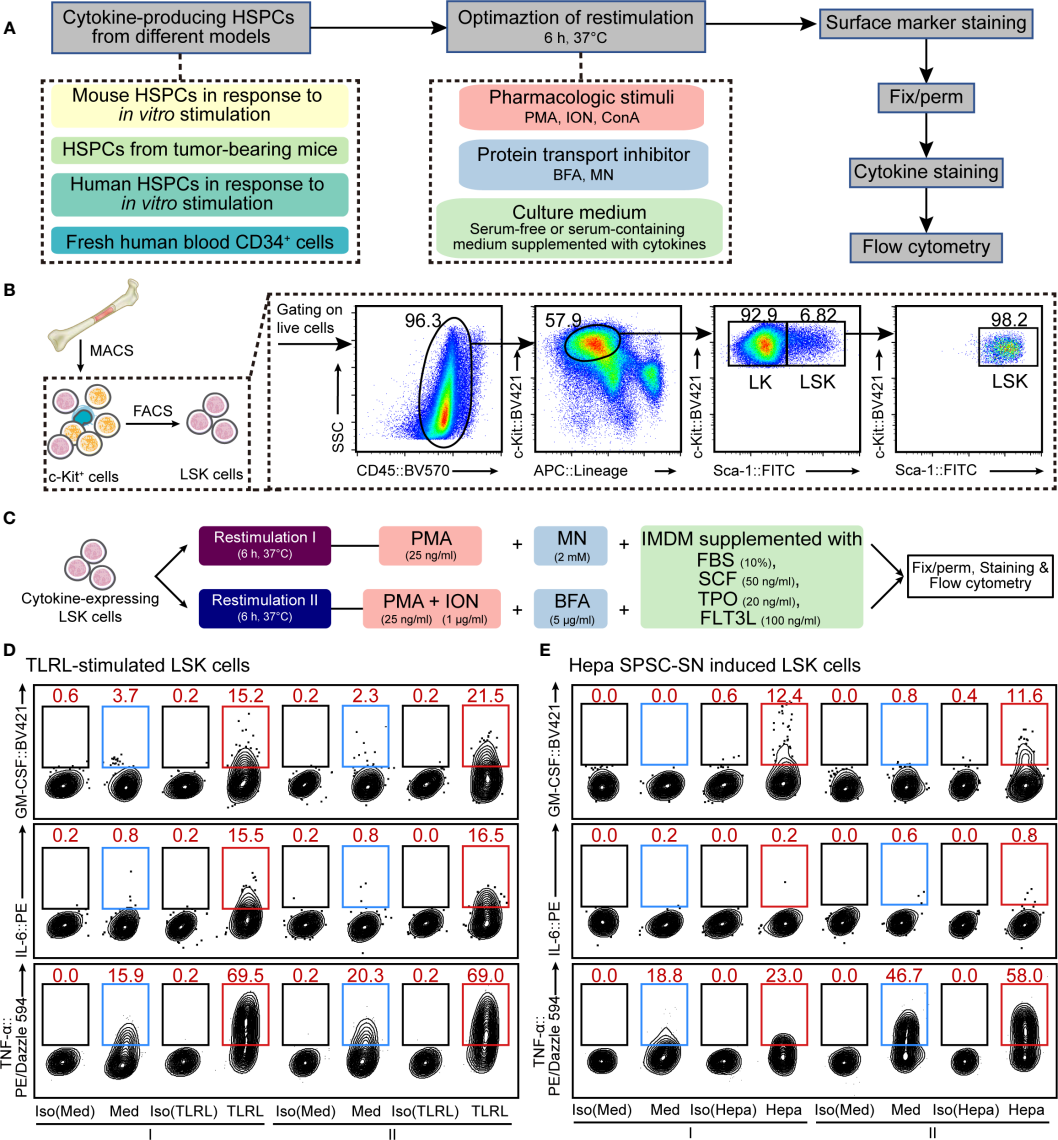
Optimization of cellular restimulation strategy. **(A)** Schematic representation of the experimental approach. **(B)** Schematic representation of HSPC isolation and the flow cytometry sorting strategy. **(C)** Two restimulation strategies, combining pharmacologic stimuli, protein transport inhibitor and culture system, were selected based on the results shown in **Supplemental Figures 2–5**. **(D, E)** Representative flow cytometric analysis of cultured LSK cells exposed to LPS and Pam3CSK4 **(D)** or Hepa SPSC-SN **(E)**. The intracellular GM-CSF, IL-6 and TNF-α levels were examined. The data refer to a typical experiment out of three that generated similar results. The numbers in the flow cytometry plots indicate the proportions of gated cells. Naïve LSK cells were cultured in the indicated medium for 12 h **(D)** or 4 days **(E)** before restimulation. After that, cells were washed and cultured in the restimulation conditions as indicated for an additional 6 h before staining. I, restimulation I; II, restimulation II; Med, control medium (SFEM + SCF); TLRL, control medium supplemented with 100 ng/ml LPS and 1 μg/ml Pam3CSK4; Hepa, control medium supplemented with 5% (v/v) Hepa mice-derived SPSC-SN; BV421, Brilliant Violet 421; PE, phycoerythrin.

A total of four cytokine-expressing HSPC models were used in this study (**Figure 1A**). For HSPC *in vitro* induction, an acute TLR stimulation model (13) and a tumor-associated splenic niche “reprograming” model (18) were constructed as previously reported. Briefly, murine and human HSPCs were cultured in StemSpan SFEM (Stem Cell Technologies, Canada) supplemented with recombinant mouse or human stem cell factor (SCF, 50 ng/ml; PeproTech Inc., USA). For TLR stimulation, murine BM LSK cells or human CD34^+^ cells were exposed to lipopolysaccharide (LPS, 100 ng/ml; Sigma-Aldrich, USA) and Pam3CSK4 (1 μg/ml; InvivoGen, USA) for 12 h. For splenic niche education, mouse splenic stromal cells were isolated from tumor-free or hepatoma-bearing mice using an enzyme-based digestion method (1 μg/ml collagenase I, 50 μg/ml hyaluronidase, 30 μg/ml collagenase XI, 50 μg/ml DNase I; Sigma-Aldrich). Lin^-^Ter-119^-^CD45^-^CD117^-^ splenic stromal cells were isolated by MACS, using biotin conjugated antibodies (BioLegend, USA) and anti-biotin microbeads (Miltenyi Biotec, Germany). Splenic stromal cell supernatant (SPSC-SN) was harvested from a 48-h culture of 5 × 10^6^/ml splenic stromal cells in RPMI 1640 supplemented with 10% fetal bovine serum (FBS, Thermo Fisher Scientific, USA). Naïve BM LSK cells were cultured in StemSpan SFEM supplemented with 50 ng/ml SCF and 5% SPSC-SN (v/v) for 4 d to induce a distinct subset of cytokine-expressing LSK cells that were found in the spleen of tumor-bearing mice, as previously reported (13). All cultures were incubated at 37°C in a 5% CO_2_ humidified atmosphere.

#### Restimulation

Freshly isolated or cultured cytokine-expressing HSPCs were subject to a short-term *in vitro* restimulation, which is key to successful intracellular cytokine staining. In this study, we tested different combinations of pharmacologic stimuli, protein transport inhibitors, and culture systems (**Figure 1** and **Supplemental Figures 1–5**, see *RESULTS* section for details). Details on reagents are provided in **Supplemental Table 2**. Two candidate conditions were selected for further validation and comparison: 1) restimulation I, HSPCs were cultured in Iscove’s modified Dulbecco’s medium (IMDM, Thermo Fisher Scientific) supplemented with 10% FBS, 50 ng/ml SCF, 20 ng/ml thrombopoietin (TPO), 100 ng/ml Fms-related tyrosine kinase 3 ligand (FLT3L), 25 ng/ml phorbol myristate acetate (PMA), and 2 µM monensin (MN) for 6 h; 2) restimulation II, HSPCs were cultured in IMDM supplemented with 10% FBS, 50 ng/ml SCF, 20 ng/ml TPO, 100 ng/ml FLT3L, 25 ng/ml PMA, 1 μg/ml ionomycin (ION), and 5 μg/ml brefeldin A (BFA) for 6 h. All cultures were incubated at 37°C in a 5% CO_2_ humidified atmosphere.

#### Surface Staining

After restimulation, cells were washed and suspended in 100 µl of staining buffer (PBS supplemented with 0.2% bovine serum albumin). To block mouse Fc receptors, cells were incubated with 1 µg 2.4G2 antibody (BD Fc Block, BD Biosciences, USA) for 15 minutes at 4°C. Fc receptors on human cells were pre-blocked by incubating cells with 10% normal human AB serum. The cells were then washed and stained with the appropriate amounts of fluorochrome-conjugated antibodies specific for cell surface antigens in 100 µl of staining buffer at 4°C for 30 minutes in the dark.

#### Fixation and Intracellular Staining

After surface staining, cells were washed and thoroughly resuspended in 250 µl Fixation/Permeabilization solution (BD Biosciences) for 20 minutes at 4°C. Then, the cells were washed two times in BD Perm/Wash buffer (BD Biosciences) and pelleted before intracellular staining. All intracellular cytokine staining experiments were controlled using isotype antibodies, i.e. antibodies of the same antibody class conjugated to the same fluorochrome. The fixed/permeabilized cells were stained with fluorochrome-conjugated anti-cytokine antibodies or isotype control antibodies in 100 µl of BD Perm/Wash buffer at 4°C for 30 minutes in the dark. Different cytokines were stained simultaneously in the same sample. To stop intracellular staining, cells were washed 2 times with BD Perm/Wash buffer and resuspend in staining buffer prior to flow cytometric analysis.

#### Data Acquisition and Analysis

Data were acquired on a CytoFLEX S flow cytometer (Beckman Coulter, USA) and analyzed with CytExpert (Beckman Coulter) and FlowJo software (Tree Star, USA). FlowSOM analysis (20) was performed using Cytobank (Cytobank, Inc., USA).

### *In Vitro* Analysis of Myeloid Differentiation

To analyze myeloid differentiation, sorted LSK cells (1,000 cells per 100 μl medium) were cultured in IMDM supplemented with 10% FBS, 50 ng/ml SCF, 100 ng/ml LPS, and 1 μg/ml Pam3CSK4 in the presence of various cytokine-neutralizing antibodies. Antibody concentrations were as follows: anti-IL-6 (1 mg/ml; BioLegend, USA), anti-GM-CSF (5 mg/ml; eBioscience, USA), and anti-TNF-α (5 mg/ml; eBioscience, USA). Cells were analyzed on day 4 by flow cytometry to determine the percentage and number of HSPCs and descendant cells.

### ELISA

Levels of GM-CSF, IL-6 or TNF-α in conditioned medium were determined with commercial high-sensitivity ELISA Kits (human GM-CSF, Cat. No. EK163HS, Multi-Science, China; human IL-6, Cat. No.88-7066, Invitrogen; human TNF-α, Cat. No. 88-7346, Invitrogen; mouse GM-CSF, Cat. No. EK263HS, Multi-Science; mouse IL-6, Cat. No. EK206HS, Multi-Science; and mouse TNF-α, Cat. No. EK282HS, Multi-Science) according to the manufacturer’s instructions. To measure cytokine secretion, murine or human HSPCs were cultured in round-bottom 96-well plates with 200 μl SFEM. The culture supernatant of TLRL-stimulated HSPCs was collected after a 12 h-culture, while the culture supernatant of Hepa SPSC-SN–stimulated HSPCs was collected after a 4 d-culture, as described above. Cytokine levels in culture supernatants were measured in triplicate.

### ELISPOT

The secretion of cytokines from the different CD34^+^ subsets (CD34^+^CD38^-^ and CD34^+^CD38^-^ cells) was detected by the enzyme-linked immunosorbent spot (ELISPOT) technique. GM-CSF (Cat. No. 3480-4APW-2; Mabtech, Sweden), IL-6 (Cat. No. 3460-4APW-2; Mabtech), and TNF-α (Cat. No. 3512-4APW-2; Mabtech) were measured according to the manufacturer’s instructions. CD34^+^CD38^-^ cells or CD34^+^CD38^+^ cells from cord blood mononuclear cells were added to the plates in triplicate with either IMDM-based complete medium (with FBS, SCF, TPO, and FLT3L) or this medium supplemented with LPS/Pam3CSK4 and cultured for 20 h at 37°C with 5% CO_2_. Plates were scanned and counted using an ELISPOT reader (Cellular Technology, Ltd., USA) to determine the number of spots in each well.

### Statistical Analysis

All statistical tests were two-sided. We used Student’s *t* test to compare means of the two groups. For multiple comparisons (including multiple two-group comparisons shown in the same panel), one-way or two-way ANOVA followed by Bonferroni’s correction (only two groups were compared), Dunnett’s test (all groups were compared with one control group), or Tukey’s multiple comparisons test (all groups were compared with each other). Statistical tests were performed with GraphPad Prism 8 (GraphPad Software, USA). Error bars indicate the mean and SEM. *P* values less than 0.05 were considered significant.

## Results

### Optimization of *In Vitro* Restimulation

A key aspect of intracellular cytokine staining is cell restimulation, which supports cells to continue producing cytokines *in vitro* while sequestering cytokines within the cell. HSPCs often produce low to undetectable amounts of cytokines *in vitro* or *ex vivo* (13, 18), so detection typically requires activation using pathogenic, mitogenic, or pharmacologic stimuli along with the inclusion of a protein transport inhibitor to sequester cytokines. Moreover, HSPCs are hypersensitive to environmental changes and thus need delicate niche factors to maintain their functionality (21–23). Therefore, to develop a protocol for the detection of intracellular cytokine production, we assessed different strategies that combined cellular restimulation and culture systems with intracellular cytokine labeling (**Figure 1A**).

First, we tested the effect of typical agents used for T cell restimulation (15, 16) on murine HSPCs. Lin^low/-^Sca-1^+^c-Kit^high^ (LSK) cells, a heterogeneous mixture of HSCs and multipotent progenitors (MPPs), were isolated from mouse BM (**Figure 1B**) and cultured in SFEM supplemented with SCF. Cells were activated with LPS and Pam3CSK4, a combination of TLR ligands (TLRLs) that was reported (13) to effectively stimulate LSK cells to produce cytokines, including GM-CSF, IL-6, and TNF-α (**Supplemental Figure 1**). LSK cells were restimulated with PMA, ION, or ConA in combination with protein transport inhibitors such as BFA or MN for a period of 6 h before intracellular staining and flow cytometric analysis (See *MATERIALS AND METHODS* for more details). The combination of PMA, ION, and BFA (PIB combination) and, to a lesser extent, PMA plus MN (PM combination) effectively maintained all three cytokines in activated mouse LSK cells (**Supplemental Figure 2**). Besides this acute infection model, similar results were obtained from a tumor-related model in which LSK cells were exposed to the culture supernatant of splenic stromal cells isolated from hepatoma-bearing mice (Hepa SPSC-SN; **Supplemental Figure 3**). This allowed a “reprograming” of the HSPC function, inducing a distinct subset of cytokine-expressing HSPCs that are enriched in the spleen of tumor-bearing mice (18). A 4-day education by Hepa SPSC-SN induced marked up-regulation of cytokine expression in cultured HSPCs. Consistent with the observation in the acute TLR-stimulated HSPCs, PIB and PM combinations were effective in maintaining HSPC functionality (**Supplemental Figure 3C**).

To fully support cell restimulation and cytokine production, the components of the medium, such as the Ca^2+^ concentration, nutrient supplements, and supportive niche factors, can be critical. We then attempted to optimize the culture system for restimulation. Both serum-containing complete medium (IMDM supplemented with 10% FBS) and SFEM, along with medium that included SCF, TPO and FLT3L, were tested. Overall, IMDM with 10% FBS was superior to SFEM for supporting mouse HSPC cytokine expression in both TLRL stimulation (**Supplemental Figure 4**) and tumor-associated splenic niche education models (**Supplemental Figure 5**). The presence of SCF, TPO, and FLT3L enhanced the expression of GM-CSF and TNF-α in activated LSK cells, but the inclusion of these recombinant cytokines in culture only marginally impacted on IL-6 expression (**Supplemental Figures 4** and **5**). Based on these results, we concluded that culturing HSPCs in IMDM-based complete medium (with FBS, SCF, TPO, and FLT3L) combined with either PM (collectively referred to as restimulation I hereafter) or PIB (collectively referred to as restimulation II hereafter) might be ideal for supporting HSPC cytokine expression *in vitro* (**Figure 1C**).

### Measurement of Cytokine Expression in LSK Cells Upon Stimulation

Using the above strategies, we measured the proportion of LSK cells that were capable of producing GM-CSF, IL-6, or TNF-α in response to immunological stresses. Few LSK cells expressed detectable GM-CSF and IL-6 in the absence of TLRLs or Hepa SPSC-SN, supporting the notion that the production of these cytokines by LSK cells is stimulation-dependent (**Figure 1D** and **Supplemental Figure 6**). Under TLRL stimulation, the average percentages of GM-CSF–producing and IL-6–producing LSK cells were approximately 15%–20% (**Figure 1D** and **Supplemental Figure 6A**), consistent with data obtained with microfluidic-based technology (13). A 4-day culture with Hepa SPSC-SN also increased GM-CSF expression but not IL-6 expression in LSK cells (**Figure 1E** and **Supplemental Figure 6B**). Notably, TNF-α was produced by approximately 15% of LSK cells cultured in control medium, and a combination of LPS and Pam3CSK4 increased that percentage to 70%, suggesting that the majority of LSK cells were capable of producing this cytokine under stress conditions (**Figure 1D**). This finding was further supported by the observation that TNF-α was detected in over 55% of LSK cells that were exposed to Hepa SPSC-SN (**Figure 1E**). Thus, our improved protocol provides a valuable approach for measuring cytokine production in HSPCs that complements existing methods and enables further mechanistic studies.

### Functional Significance of HSPC-Produced Cytokines in Regulating Myelopoiesis *In Vitro*

**Figure 2:**
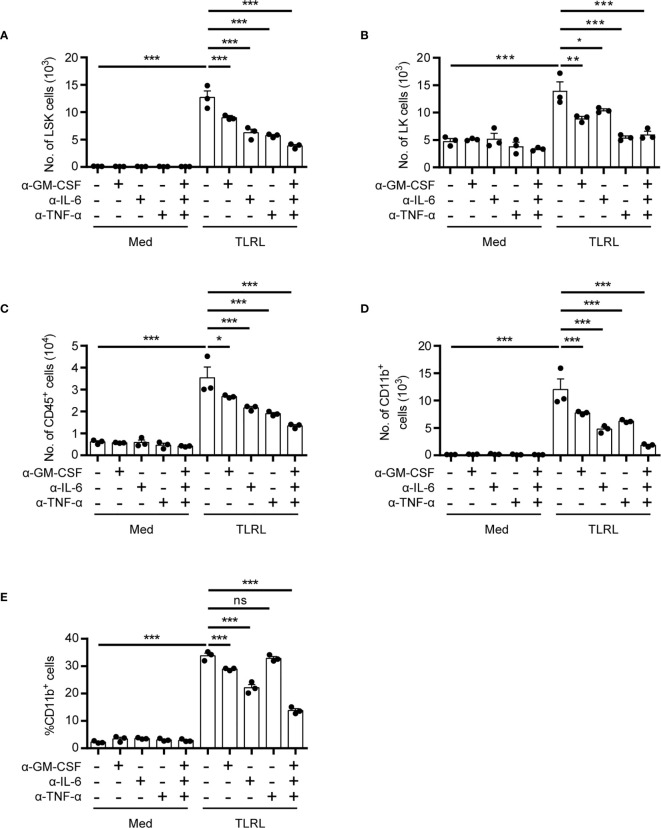
Functional impact of HSPC-produced cytokines on myelopoiesis in response to TLR stimulation. Analysis of the *in vitro* myelopoiesis of LSK cells by flow cytometry. Sorted LSK cells (1,000 cells per 100 μl medium) were cultured in IMDM supplemented with FBS (10%, v/v), SCF (50 ng/ml), with or without the addition of LPS (100 ng/ml), Pam3CSK4 (1 mg/ml), and the indicated cytokine-neutralizing antibodies. Cells were analyzed on day 4 by flow cytometry. Numbers of LSK cells **(A)**, LK cells **(B)**, CD45^+^ total immune cells **(C)**, and the number **(D)** and percentage **(E)** of CD11b^+^ myeloid cells were examined. Columns represent the mean of results from three separate experiments, and error bars represent the SEM. *P < 0.05, **P < 0.01, ***P < 0.001; ns, not significant, by 1-way ANOVA followed by Dunnett’s test.

Despite their rarity, the close proximity of HSPCs in the stem cell niche may provide a unique advantage for rapid autocrine- or paracrine-mediated hematopoiesis. To assess the functional significance of HSPC-produced cytokines for myelopoiesis induced by TLRL stimulation, we examined the effect of neutralizing antibodies on LSK cell differentiation *in vitro*. Interestingly, the neutralization of GM-CSF, IL-6, or TNF-α reduced the number of LSK cells, and blocking all three signals resulted in an even more significant decrease in the LSK cell number, suggesting that these cytokines promote HSPC proliferation and/or survival (**Figure 2A**). In addition, all three cytokines contributed to the differentiation of LSK cells to Sca-1^-^ LK myeloid progenitor cells (**Figure 2B**) and increased the overall numbers of CD45^+^ immune descendants (**Figure 2C**). However, the neutralization of IL-6 had a stronger inhibitory effect on the generation of CD11b^+^ myeloid cells than the neutralization with anti–GM-CSF or anti–TNF-α antibodies (**Figures 2D, E**). These results confirmed our flow cytometric data showing that HSPCs are capable of producing inflammatory cytokines to coordinate rapid, stress-induced myelopoiesis in an autocrine or paracrine fashion.

### Validation of Restimulation Conditions Using Freshly Isolated Mouse HSPCs

**Figure 3:**
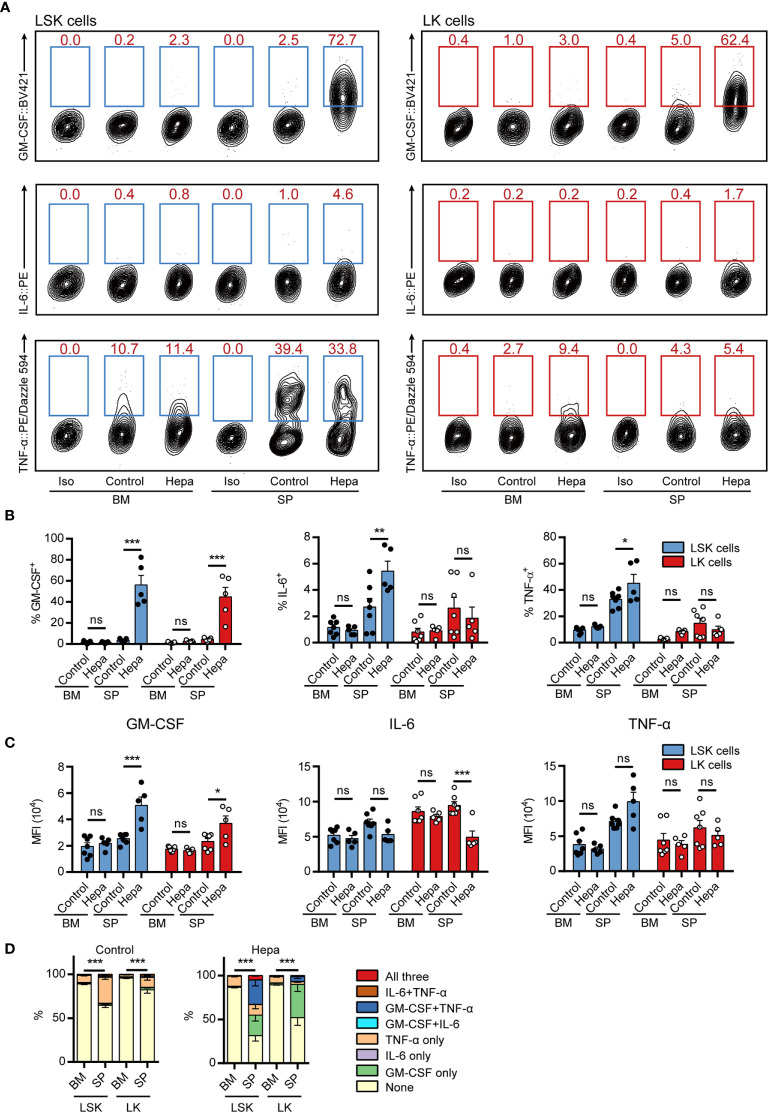
Measuring intracellular cytokine levels in freshly isolated HSPCs from hepatoma-bearing mice with restimulation II. **(A)** Representative flow cytometric analysis of intracellular GM-CSF, IL-6 and TNF-α in freshly isolated LSK cells (left) and LK cells (right) from the BM and spleen of control or Hepa mice. The numbers in the flow cytometry plots indicate the proportions of gated cells. The data refer to a typical experiment out of three that generated similar results. Iso, isotype antibody control. **(B)** The percentages of GM-CSF^+^, IL-6^+^, and TNF-α^+^ cells in LSK and LK cells (*n* = 5–7 per group). *P < 0.05, **P < 0.01, ***P < 0.001; ns, not significant, by 2-way ANOVA followed by Tukey’s test. **(C)** The median fluorescence intensity (MFI) of GM-CSF, IL-6, and TNF-α in the corresponding cytokine-positive cells (*n* = 5–7 per group). Data are shown as the mean ± SEM. *P < 0.05, **P < 0.01, ***P < 0.001; ns, not significant, by 2-way ANOVA followed by Tukey’s test. **(D)** Polyfunctionality of LSK and LK cells from the BM and spleen of control or Hepa mice (*n* = 5–7 per group). Data are shown as the mean ± SEM. ***P < 0.001, by a two-way ANOVA with interaction.

Having established two potential restimulation strategies, we next examined whether the intracellular staining methods could be applied to assess cytokine expression in freshly isolated BM and splenic (SP) HSPCs from tumor-bearing mice. Comparing restimulation I and II, we found that restimulation II was superior for maintaining cytokine expression in freshly isolated HSPCs (**Figures 3A–C**, compared with **Supplemental Figures 7A–C**). Consistent with our previous findings, GM-CSF was primarily expressed by splenic HSPCs from mice bearing orthotopic Hepa1-6 hepatoma (hereafter referred to as Hepa mice) but not by those from tumor-free control mice or by HSPCs from the BM of tumor-bearing mice (**Figures 3A–C**). Using the present protocol, we found that more than 50% of Hepa splenic LSK cells and approximately 40% of LK cells expressed GM-CSF. These percentages were significantly higher than those obtained using our previous method (18), indicating that this cytokine was upregulated in the majority of HSPCs residing in the spleen of tumor-bearing mice.

Collectively, 30%–40% of splenic LSK cells expressed TNF-α in a tumor-independent manner (**Figure 3D**). Except for these cells, few HSPCs from tumor-free mice or from the BM of Hepa mice expressed any of these three cytokines. In contrast, a significant proportion of LSK cells from the spleen of Hepa mice simultaneously expressed two or more of these cytokines. Approximately 30% of Hepa splenic LSK cells expressed both GM-CSF and TNF-α and 5% of LSK cells simultaneously expressed GM-CSF, IL-6, and TNF-α (**Figure 3D**). These data demonstrate that restimulation II, i.e., *ex vivo* culture of HSPCs in IMDM-based complete medium (with FBS, SCF, TPO, and FLT3L) supplemented with PIB for a short period enables the simultaneous intracellular staining of multiple cytokines in mouse HSPCs.

### Validation of Intracellular Cytokine Staining in Human HSPCs

**Figure 4:**
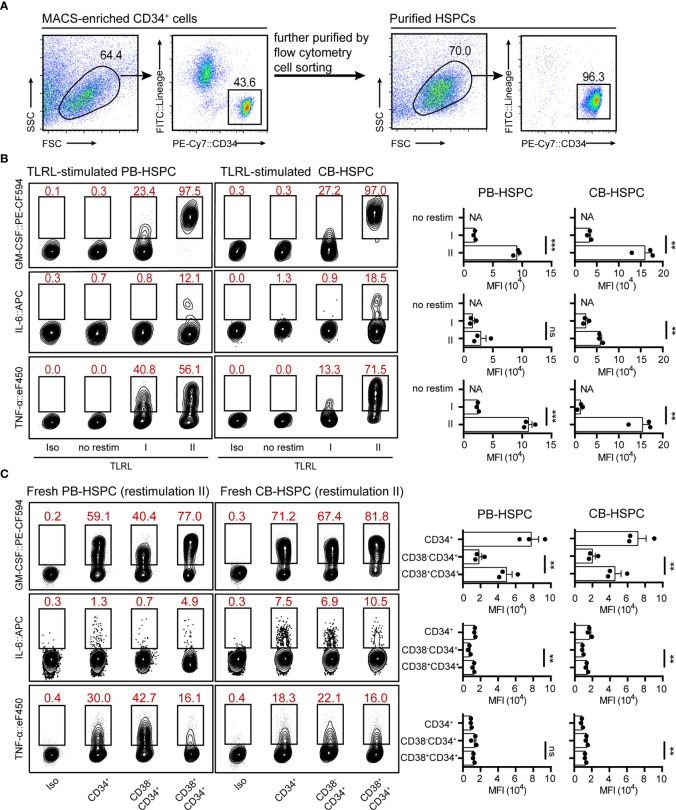
Cytokine expression levels in stimulated and freshly isolated human HSPCs. **(A)** The gating strategy and the population purity after flow cytometry cell sorting. **(B)** Representative flow cytometry plots showing GM-CSF, IL-6 and TNF-α levels in TLRL-stimulated CD34^+^ cells from PB or CB. To obtain a sufficient number of HSPCs, lin^-^CD34^+^ cells were first expanded in SFEM supplemented with 50 ng/ml SCF. On day 3, these cells were exposed to LPS and Pam3CSK4 for 12 h, and then cells were harvested for flow cytometry analysis (with restimulation II). The data refer to a typical experiment out of three that generated similar results. **P < 0.01, ***P < 0.001; ns, not significant, by Student’s *t* test. **(C)** Representative flow cytometry plots showing GM-CSF, IL-6 and TNF-α levels in freshly isolated human blood-derived HSPCs (*n* = 3 per group). Cells isolated from fresh blood were treated with restimulation II before flow cytometric analysis. The data refer to a typical experiment out of three that generated similar results. **P < 0.01; ns, not significant, by 1-way ANOVA followed by Tukey’s test. The numbers in the flow cytometry plots indicate the proportions of gated cells. APC, allophycocyanin; eF450, eFluor 450; FITC, fluorescein isothiocyanate; NA, not available.

To test whether the optimized protocol can also be applied for human HSPC staining, we examined cytokine expression in TLRL-stimulated human HSPCs. Human lin^-^CD34^high^ HSPCs isolated from adult peripheral blood (PB-HSPCs) or neonatal cord blood (CB-HSPCs) were sorted and examined (**Figure 4A**). Consistent with the findings in freshly isolated mouse HSPCs, restimulation II was superior to restimulation I for maintaining cytokine expression in short-term cultured PB-HSPCs and CB-HSPCs (**Figure 4B** and **Supplemental Figure 8A**). Thus, we used restimulation II in later experiments. To our surprise, a significantly higher proportion of human HSPCs expressed GM-CSF, IL-6, or TNF-α in response to TLRL stimulation (**Figure 4B**) than mouse HSPCs (**Figure 3**). Among the 3 cytokines studied, GM-CSF was the most highly induced and was expressed by over 95% of PB-HSPCs and over 90% of CB-HSPCs.

These data indicate that restimulation II is also suitable for human HSPC staining. However, it was not clear whether the expression of cytokines was induced by TLRLs or whether these cytokines were constitutionally expressed by human circulating HSPCs. Therefore, we examined cytokine expression in freshly isolated PB-HSPCs and CB-HSPCs. Approximately half of PB-HSPCs and over 70% of CB-HSPCs expressed GM-CSF (**Figure 4C** and **Supplemental Figure 8B**). Human circulating lin^-^CD45^+^CD34^+^ cells represent a heterogeneous population comprising CD34^+^CD38^-^ early progenitors and CD34^+^CD38^+^ committed progenitors. GM-CSF^+^ HSPCs were further enriched in the CD34^+^CD38^+^ population, suggesting that this myeloid differentiation signal is constitutionally expressed in the vast majority of circulating committed progenitors. In contrast, TNF-α was enriched in CD34^+^CD38^-^ early HSPCs, and IL-6 was expressed only by a small fraction of circulating HSPCs, demonstrating different cytokine expression patterns.

**Figure 5:**
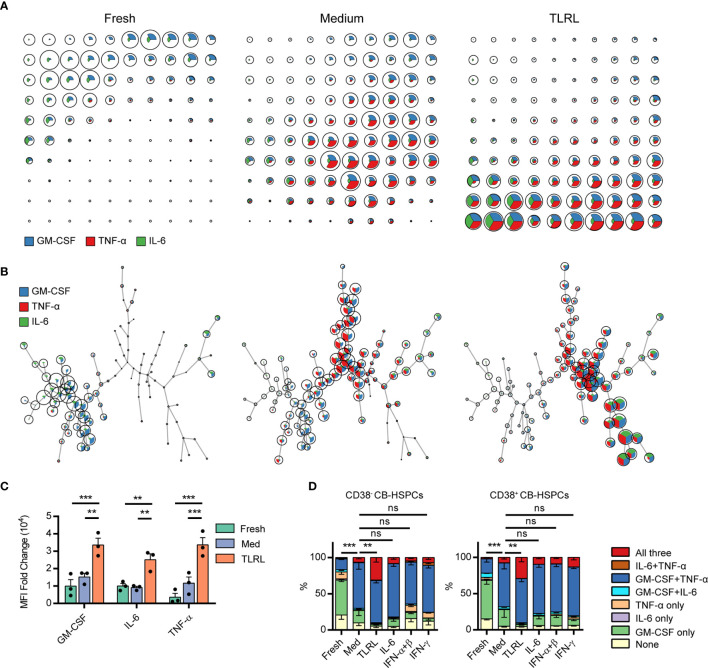
Polyfunctional cytokine production capability of human CD34^+^ cells. **(A, B)** FlowSOM was used to train a self-organizing map (SOM) for cytokine staining in freshly isolated, cultured without stimulation, and TLRL-stimulated CB-derived CD34^+^ cells. The SOM results are shown on grids **(A)** or on minimal spanning trees **(B)**. Each cell was classified to the nearest node, which is coded as a pie chart with information about the expression of the three cytokines. The size of the nodes is relative to the percentage of cells presented in each cluster. Each node is represented by a star chart indicating the relative mean fluorescence intensity values of each cytokine; the height of each sector indicates the intensity; if the part reaches the border of the circle, the cells have maximal expression for that cytokine. **(C)** MFI values of each cytokine in freshly isolated, cultured without stimulation, and TLRL-stimulated CB-derived CD34^+^ cells. The data refer to a typical experiment out of three that generated similar results. Data are shown as the mean ± SEM. **P < 0.01 and ***P < 0.001, by 2-way ANOVA followed by Tukey’s test. **(D)** Polyfunctionality of CB-HSPCs stimulated for 12 h with various stimuli. The data refer to a typical experiment out of three that generated similar results. Data are shown as the mean ± SEM. **P < 0.01, ***P < 0.001; ns, not significant, by a two-way ANOVA with interaction.

A 12-h *in vitro* culture, especially with TLR stimulation, significantly expanded the population of multipotent cytokine producers in the CB-HSPC compartment. To provide a clear overview of cytokine expression in fresh, cultured, and TLRL-stimulated CB-HSPCs, a self-organizing map (SOM) was built and visualized with a grid (**Figure 5A**) or with a minimal spanning tree (**Figure 5B**). Exposure to TLRLs dramatically enhanced the cytokine production capability of CB-HSPCs in terms of both quantity and breadth. TLRLs markedly upregulated cytokine expression in HSPCs (**Figure 5C**). Furthermore, over 90% of TLR-activated CB-HSPCs were capable of simultaneously producing GM-CSF and TNF-α, one-third of which were also positive for IL-6 staining (**Figure 5D**). TLRLs (LPS + Pam3CSK4) were superior to common proinflammatory cytokines, including IL-6, type I interferons (IFN-α + IFN-β), and IFN-γ, in inducing multipotent cytokine producers in the CB-HSPC compartment. Together, these data suggest that circulating HSPCs are potent cytokine producers in response to danger signals.

### Cytokine Production and Secretion by Human HSPCs

**Figure 6:**
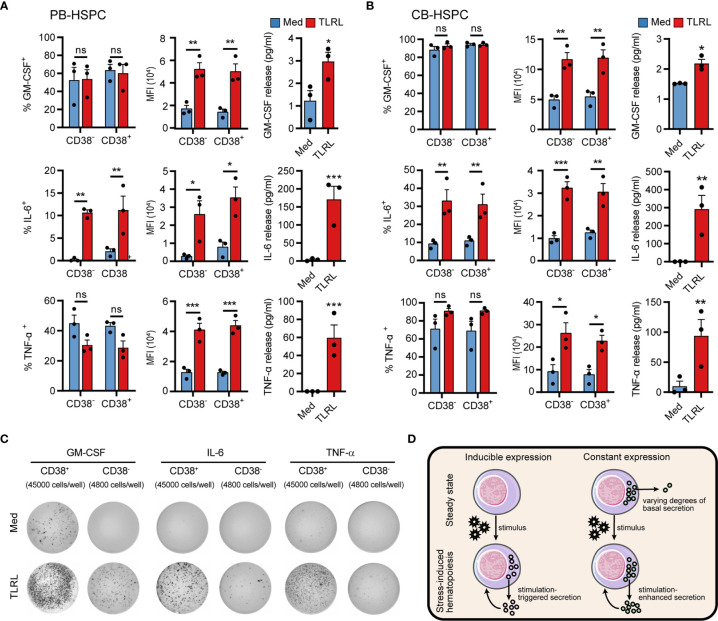
Cytokine production and secretion by PB- or CB-derived HSPCs. **(A, B)** Adult PB- **(A)** or neonatal CB-derived **(B)** CD34^+^ HSPCs were either left untreated or stimulated with LPS and Pam3CSK4 for 12 h. The percentages of cell expressing GM-CSF, IL-6 and TNF-α were determined by flow cytometry. MFI of the cytokine signals in the corresponding positive cells are shown. The secretion of cytokines in the supernatant was quantified by ELISA, with 20,000 CD34^+^ cells in 200 µl medium. Data are shown as the mean ± SEM. *P < 0.05, **P < 0.01, and ***P < 0.001; ns, not significant, by 2-way ANOVA followed by Bonferroni’s test or Student’s *t* test. **(C)** The number of cytokine-expressing HSPCs were measured by ELISPOT. CD34^+^CD38^-^ cells (4,800 cells/well) or CD34^+^CD38^-^ cells (45,000 cells/well) were incubated for 20 h in ELISPOT plates in the presence or absence of LPS and Pam3CSK4. The data refer to a typical experiment out of three that generated similar results. **(D)** Summary of two different regulatory modes of cytokine production in HSPCs.

The synthesis and secretion of cytokines is an integral process that is driven in a stepwise manner and separately regulated by different mechanisms. To obtain a clearer picture of HSPC cytokine signaling, we examined both the intracellular production (using flow cytometry) and secretion (using ELISA and ELISPOT, see standard curves of ELISAs and a representative gating of the flow cytometry cell sorting in **Supplementary Figure 9**) of GM-CSF, IL-6, and TNF-α by human HSPCs in response to TLRLs. GM-CSF was expressed by a large proportion of unstimulated PB-HSPCs (**Figure 6A**) and CB-HSPCs (**Figure 6B**) and was constantly released at a basal level in culture. Although TLRLs hardly elevated the proportion of GM-CSF–expressing HSPCs, they markedly increased the median fluorescence intensity (MFI) and secretion of this cytokine (**Figures 6A–C**). In contrast to GM-CSF, the secretion of IL-6 and TNF-α was tightly regulated though 2%–10% and more than 40% of unstimulated circulating HSPCs expressed these cytokines, respectively. TLRLs significantly increased the proportion of HSPCs expressing IL-6 but not TNF-α while this stimulation effectively triggered the rapid release of both of these cytokines. These data suggest that there are distinct modes of cytokine production regulation in HSPCs (**Figure 6D**): 1) inducible expression and stimulus-dependent secretion (e.g., IL-6 in human circulating HSPCs) and 2) constant expression with varying degrees of basal secretion and stimulus-triggered rapid release (e.g., GM-CSF and TNF-α in human circulating HSPCs).

## Discussion

HSPCs can produce a plethora of cytokines that adaptively regulate stress-induced hematopoiesis in an autocrine or paracrine manner. However, measuring cytokine production in HSPCs has been difficult. In this study, we optimized a flow cytometric protocol to simultaneously measure multiple cytokines in murine and human HSPCs at the single-cell level. This method is reproducible and valid, as our experiments showed that it was effective and applicable for both freshly isolated and cultured murine and human HSPCs from different models. This technique, combined with the multiparameter capability and the high throughput nature of the instrumentation, supplements existing methods for measuring cytokine release and gives enormous support for studying HSPC cytokine expression profiles.

Flow cytometry is a leading technique for measuring intracellular protein levels, such as transcription factors and signal transduction molecules, or those that are usually secreted, such as cytokines and chemokines (15). However, unlike proteins that are expressed constitutively, cytokine expression usually requires cell restimulation, as is the case for T cells (16). Here, we considered several factors when optimizing restimulation conditions. First, we found that stimulation with PMN and ION, which activate the protein kinase C/nuclear factor κB (NF-κB) and calcineurin/nuclear factor of activated T cells (NFAT) pathways, respectively, promoted the production of cytokines by HSPCs *in vitro*. These results were not entirely surprising because the cytokine production ability of HSPCs has been shown to be regulated by NF-kB activity (13, 24). In addition, NFATc2 (NFAT1) is expressed at high levels in human CD34^+^ HSPCs (25) and is required for the maintenance of steady-state hematopoiesis (26); thus, the inclusion of an NFAT agonist may be valuable for maintaining HSPC functionality *in vitro*. Second, effects of BFA and MN in blocking cytokine secretion by HSPCs were compared. One characteristic of MN is that it does not sufficiently inhibit TNF-α secretion from cells (27), which is supported by our present dataset, and we found that this characteristic of MN may also be applicable to GM-CSF. Therefore, BFA may be more suitable when these cytokines are among the factors of interest. Finally, culturing HSPCs usually requires additional supplements that mimic niche signals to support full cell functionality. We found that IMDM supplemented with 10% FBS, 50 ng/ml SCF, 20 ng/ml TPO, and 100 ng/ml FLT3L, a collection of cytokines that is often used for HSC expansion (28), was ideal for maintaining cytokine production in HSPCs during the restimulation process. Thus, these factors optimally maintain the capability of HSPCs to produce cytokines *in vitro* and enable the simultaneous staining of multiple intracellular cytokines in murine and human HSPCs.

Using the present protocol, we observed interesting cytokine expression and regulatory patterns in HSPCs. In mice, while only a small fraction of BM HSPCs expressed GM-CSF, IL-6, or TNF-α, approximately 40% of splenic LSK cells, both in tumor-free and orthotopic hepatoma-bearing mice, expressed TNF-α, suggesting niche-dependent functional heterogeneity. In addition, expression patterns vary greatly across cytokines. In contrast to the unchanged percentage of TNF-α^+^ cells, the vast majority of splenic LSK cells and, to a lesser extent, LK cells in tumor-bearing mice produced GM-CSF. These GM-CSF–expressing HSPCs were almost exclusively found in the spleen of tumor-bearing mice but not in the BM or homeostatic spleen. However, most human CD34^+^ cells from the peripheral blood of healthy adults and newborns showed positive intracellular GM-CSF staining, suggesting very different regulatory mechanisms governing the expression of this cytokine in HSPCs across species. ELISA and ELISPOT results confirmed that, regardless of the distinct expression levels in HSPCs, TLR signals remarkably enhanced the release of GM-CSF, IL-6, and TNF-α. More interestingly, we observed a non-negligible baseline level of GM-CSF secretion by human circulating HSPCs even in the steady state. A deeper understanding of the relationship between autocrine basal cytokines and the lineage potential of extramedullary HSPCs may provide novel insights into the immunosurveillance role of trafficking HSPCs and may promote HSC expansion techniques for bone marrow transplantation.

Although only three cytokines were studied in the present work, the current protocol can be adapted for measuring other or more cytokines in HSPCs. A comprehensive description of HSPC cytokine production profiles is critically important for understanding their roles in regulating steady-state and stress-induced hematopoiesis. The nature of HSPC heterogeneity on the basis of differential cytokine production or receptor expression remains an open research area for future studies. In this context, the integration of intracellular staining methods with multiparameter flow cytometry, combined with the high throughput nature of the instrumentation, provides an enormous advantage over existing methods such as ELISA and ELISPOT. This advantage can be further enhanced by applying more advanced variations of cytometry, such as mass cytometry and spectral flow cytometry, that allow for the combination of many more antibodies in a single sample, for which the current protocol should be equally applicable. However, it should be noted that the inclusion of different cytokines in the panel may require modifications of the present method. For example, it could be beneficial to re-examine the performance of BFA and MN, as well as that of fix/perm chemicals, since these reagents may have differential effects on different cytokines. Finally, it might be argued that PMA/ION represents a strong inducer of cytokine expression that may not lead to the “actual” cytokine expression by HSPCs, i.e., *in vivo* at the time point of analysis, but it is very well suited to reveal the maximal cytokine expression potential of HSPCs.

Cytokine production is one of the most important properties of immune cells, as it orchestrates a functional immune response involving both cells of adaptive and innate immunity (29). In this study, we describe a method for intracellular cytokine staining in murine and human HSPCs. This technical advance may aid in revealing the relationship between the altered cytokine production profile in HSPCs and their lineage potential and functional capacity in homeostatic and pathological conditions.

## Data Availability Statement

The raw data supporting the conclusions of this article will be made available by the authors, without undue reservation.

## Ethics Statement

The studies involving human participants were reviewed and approved by Review Board of Sun Yat-Sen University. The patients/participants provided their written informed consent to participate in this study. The animal study was reviewed and approved by IACUC of Sun Yat-Sen University.

## Author Contributions

SL, LiZ, and CW designed the experiments. SL, HL, LaZ, SY, JL, and ML performed the experiments. SL and CW analyzed and interpreted the data. H-TC provided clinical resources. LZhe and CW supported and supervised the research. SL, LiZ, and CW wrote the manuscript, and all authors contributed to editing of the manuscript. All authors contributed to the article and approved the submitted version.

## Funding

This work was supported by project grants from the National Key R&D Program of China (2017YFA0505803), the National Natural Science Foundation of China (31900640, 81730044 & 91842308), the Science and Information Technology of Guangzhou (201904020040), the Guangdong Basic and Applied Basic Research Foundation (2019A1515011991 & 2019A1515110911), and the Fundamental Research Funds for the Central Universities (171gjc32 & 20lgpy118).

## Conflict of Interest

The authors declare that the research was conducted in the absence of any commercial or financial relationships that could be construed as a potential conflict of interest.
