# Supplementary material for: Optimized Intracellular Staining Reveals Heterogeneous Cytokine Production Ability of Murine and Human Hematopoietic Stem and Progenitor Cells
Source: Front Immunol. 2021 Apr 14;12:654094. doi: 10.3389/fimmu.2021.654094 (PMC8079767; doi:10.3389/fimmu.2021.654094)
Supplement: Supplementary file 1 [file DataSheet_1.zip › Supplemental Figures and Tables.pdf]

Supplemental Materials

Optimized intracellular staining reveals heterogeneous cytokine production ability of murine and human hematopoietic stem and progenitor cells

Shufeng Luo[1,2], Huiling Lin[1], Lan Zhu[1], Hai-Tian Chen[3], Siqian Yang[1], Jinheng Li[1], Mingyu Liu[2], Limin Zheng[1,2], and Chong Wu[1*]

[1]MOE Key Laboratory of Gene Function and Regulation, School of Life Sciences, Sun Yat-sen University, Guangzhou 510275, China

[2]Collaborative Innovation Center for Cancer Medicine, State Key Laboratory of Oncology in South China, Sun Yat-sen University Cancer Center, Guangzhou 510060, China

[3]First Affiliated Hospital, Sun Yat-sen University, Guangzhou 510080, China

*Corresponding to: Chong Wu (E-mail: wuchong5@mail.sysu.edu.cn)

Supplemental Materials Inventory

1. **Supplemental Figures** (Supplemental Figures 1–9)
2. **Supplemental Tables** (Supplemental Table 1 and 2)

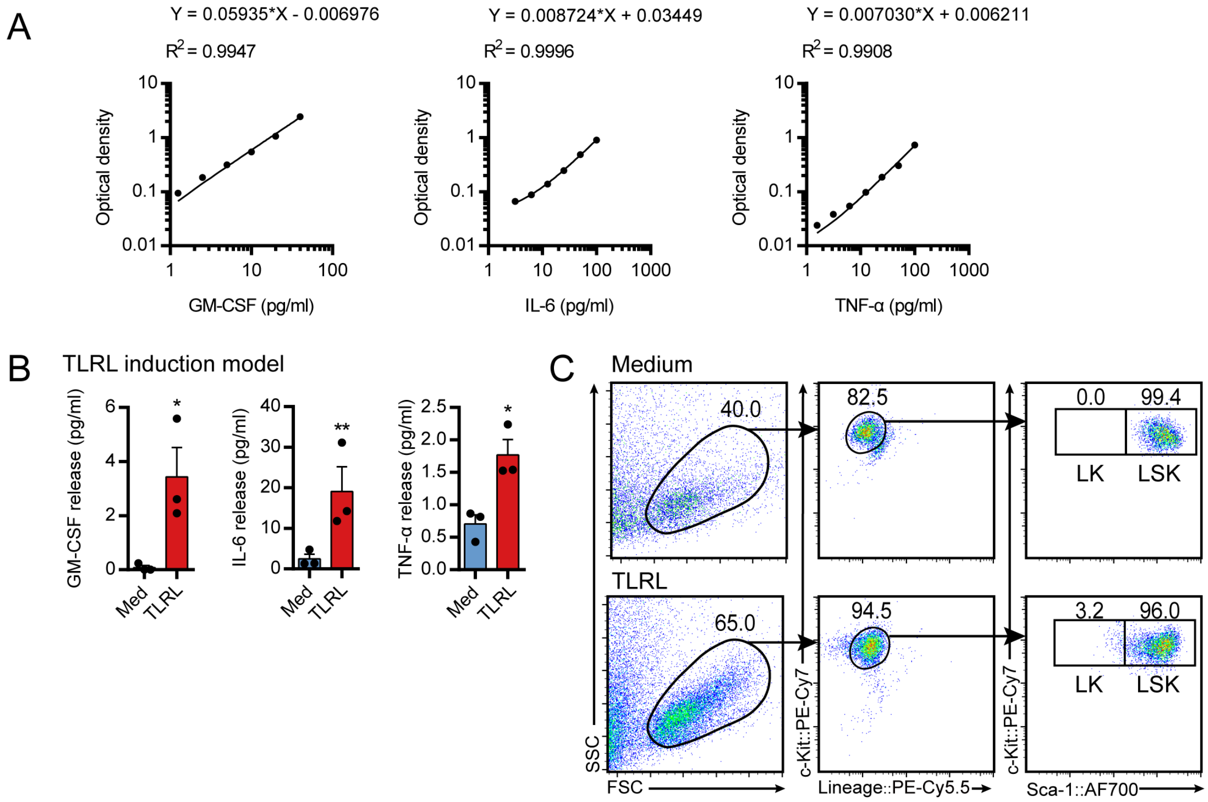

Supplemental Figure 1. Establishment of LPS/Pam3CSK4 stimulated-LSK cell model. (**A**) ELISA standard curves for measuring mouse GM-CSF, IL-6, and TNF-α. (**B**) ELISA assessment of GM-CSF, IL-6 and TNF-α in culture supernatants of LSK cells exposed to LPS and Pam3CSK4. Cultures were set up with 20,000 LSK cells in 200 μl medium for 12 h. Columns represent the mean of results from three separate experiments, and error bars represent the SEM. *$P < 0.05$ and **$P < 0.01$, by Student's *t* test. (**C**) Representative flow cytometry plots showing the phenotype of cultured LSK cells with or without exposure to LPS/Pam3CSK4. Naïve LSK cells were cultured in the indicated medium for 12 h before restimulation. Med, control medium (SFEM + SCF); TLRL, control medium supplemented with 100 ng/ml LPS and 1 μg/ml Pam3CSK4. The numbers in the flow cytometry plots indicate the proportions of gated cells.

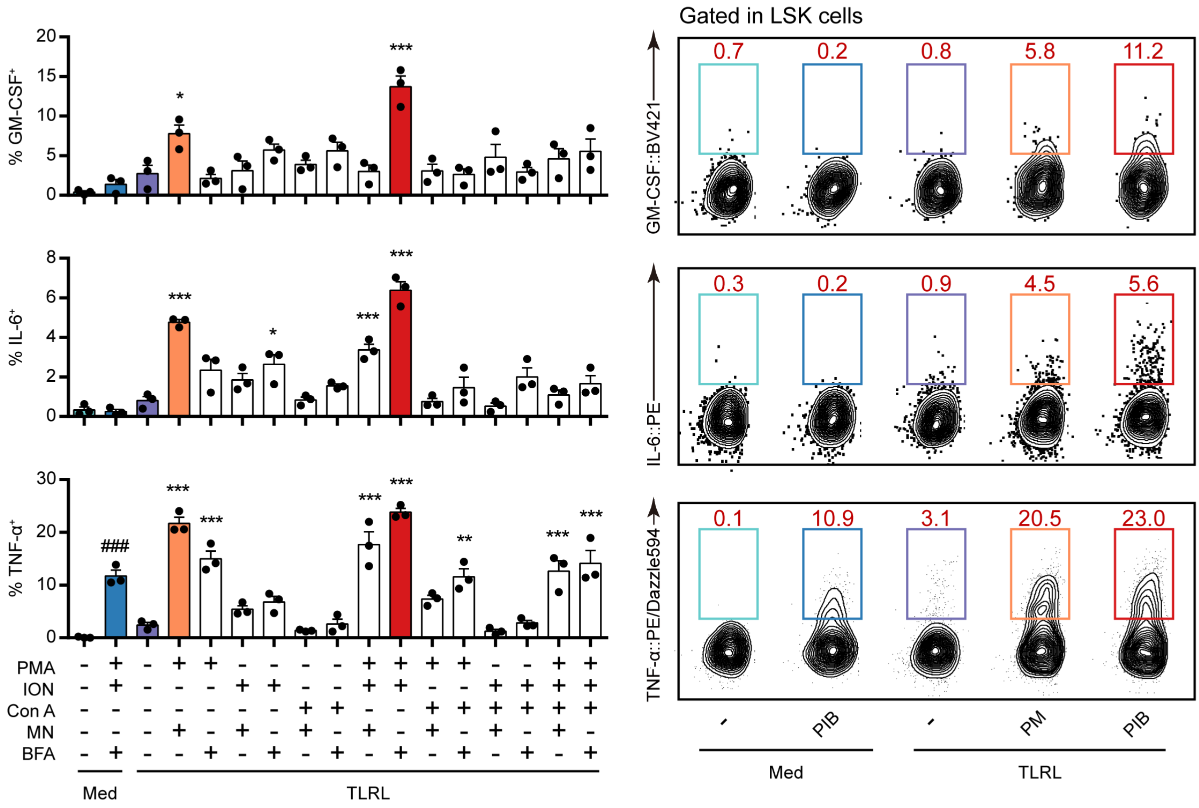

Supplemental Figure 2. Optimization of combined cellular restimulation with LPS and Pam3CSK4 stimulation. The percentages of GM-CSF$^+$, IL-6$^+$, and TNF-α$^+$ cells in TLRL-stimulated LSK cells were evaluated by flow cytometry. Naïve LSK cells were cultured in the indicated medium for 12 h. After that, cells were washed and cultured in the SFEM supplemented with 50 ng/ml SCF and indicated reagents for an additional 6 h before staining. Med, control medium (SFEM + 50 ng/ml SCF); TLRL, control medium supplemented with 100 ng/ml LPS and 1 μg/ml Pam3CSK4. PIB, the combination of PMA, ION, and BFA; PM, PMA plus MN. $^{###}$*P* < 0.001 by Student's *t* test, compared with the "no restimulation" group under Med condition. **P* < 0.05, ***P* < 0.01 and ****P* < 0.001, by 1-way ANOVA followed by Dunnett's test, compared with the "no restimulation" group under TLRL condition. Data are shown as the mean ± SEM in bar charts. The numbers in the flow cytometry plots indicate the proportions of gated cells.

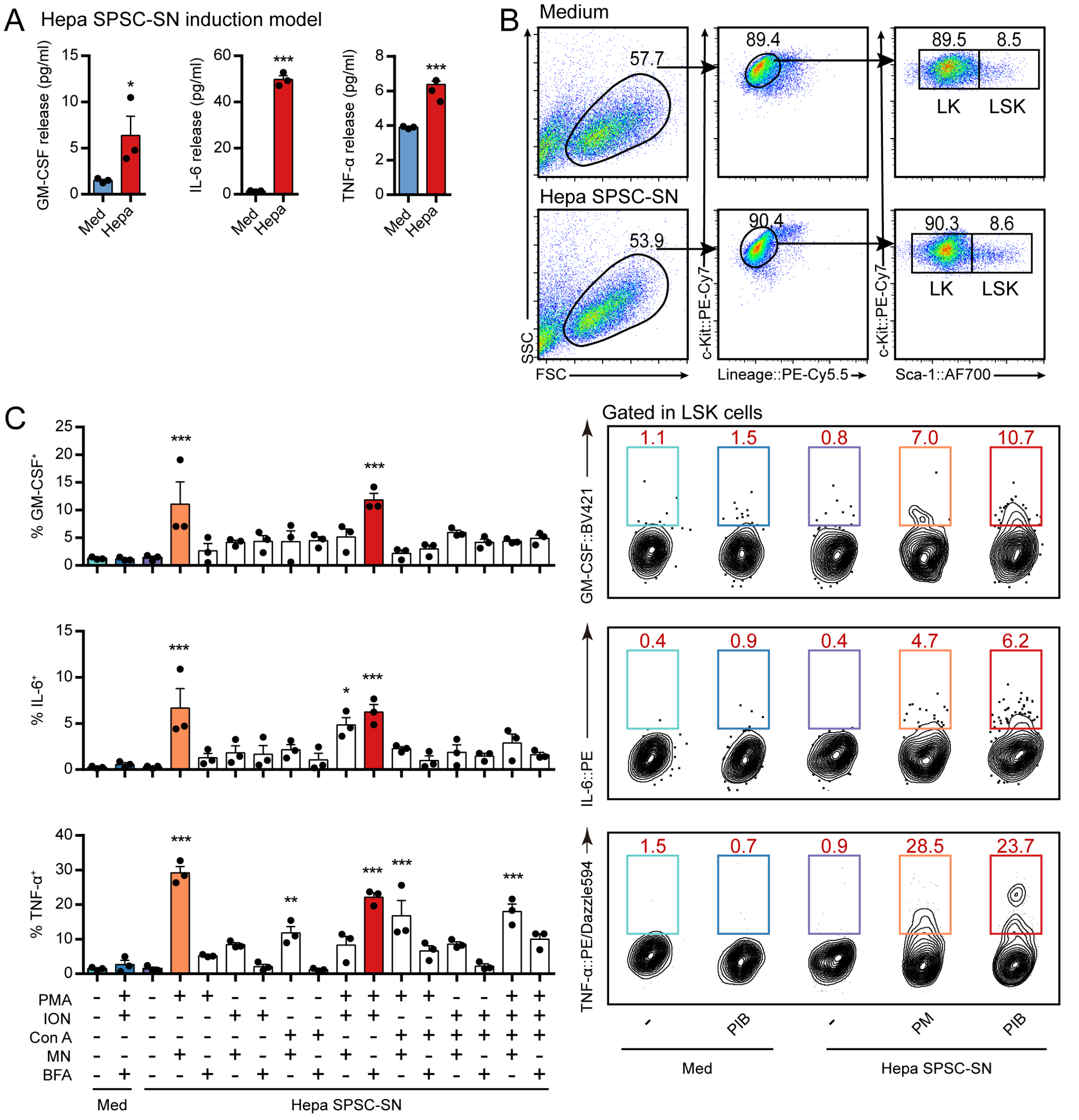

Supplemental Figure 3. Optimization of combined cellular restimulation in the *in vitro* Hepa SPSC-SN induction model. (**A**) ELISA assessment of GM-CSF, IL-6 and TNF-α in culture supernatants of LSK cells exposed to Hepa SPSC-SN. Cultures were set up with 5,000 LSK cells in 200 μl medium for 4 d. Columns represent the mean of results from three separate experiments, and error bars represent the SEM. $^*P < 0.05$ and $^{***}P < 0.001$, by Student's t test. (**B**) Representative flow cytometry plots showing the phenotype of cultured LSK cells with or without exposure to Hepa SPSC-SN. Naïve LSK cells were cultured in the indicated medium for 4 d before restimulation. Med, control medium (SFEM + SCF); Hepa SPSC-SN, control medium supplemented with 5% (v/v) Hepa SPSC-SN. The numbers in the flow cytometry plots indicate the proportions of gated cells. (**C**) The percentages of GM-CSF$^+$, IL-6$^+$, and TNF-α$^+$ cells in Hepa SPSC-SN stimulated LSK cells were evaluated by flow cytometry. Naïve LSK cells were cultured in the indicated medium for 4 days before restimulation. After that, cells were washed and cultured in the SFEM supplemented with SCF and indicated reagents for an additional 6 h before staining. Med, control medium (SFEM + 50 ng/ml

SCF); Hepa SPSC-SN, control medium supplemented with 5% (v/v) Hepa mice-derived SPSC-SN. PIB, the combination of PMA, ION, and BFA; PM, PMA plus MN. *$P < 0.05$, **$P < 0.01$ and ***$P < 0.001$, by 1-way ANOVA followed by Dunnett's test, compared with the "no restimulation" group under Hepa SPSC-SN condition. Data are shown as the mean ± SEM in bar charts. The numbers in the flow cytometry plots indicate the proportions of gated cells.

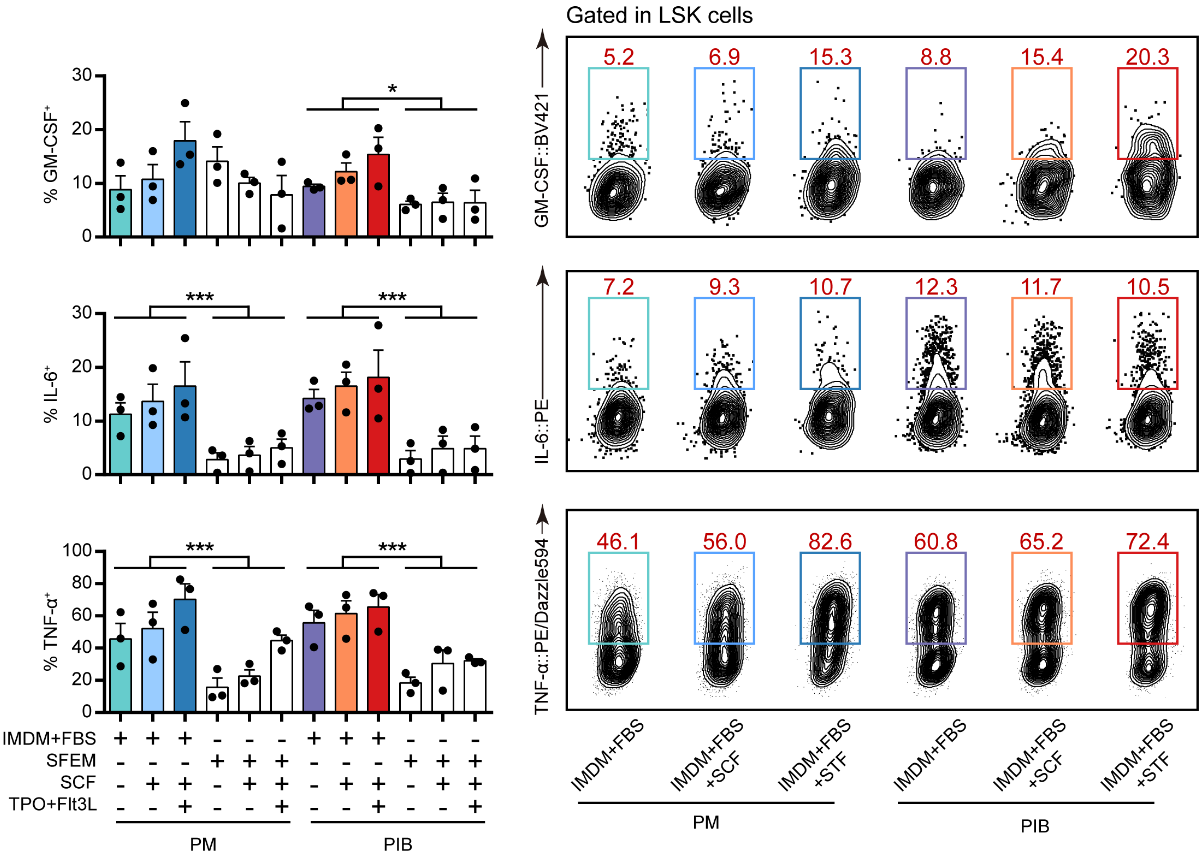

Supplemental Figure 4. Optimization of the culture system during the restimulation of TLRL-stimulated HSPCs. The percentages of GM-CSF$^+$, IL-6$^+$, and TNF-α$^+$ cells in TLRL-stimulated LSK cells were evaluated by flow cytometry. Naïve LSK cells were cultured in SFEM supplemented with 50 ng/ml SCF, 100 ng/ml LPS and 1 μg/ml Pam3CSK4 for 12 h. After that, cells were washed and cultured in the indicated medium for an additional 6 h before staining. SCF, 50 ng/ml; TPO, 20 ng/ml; FLT3L, 100 ng/ml. PIB, the combination of PMA, ION, and BFA; PM, PMA plus MN. STF, SCF + TPO + Flt3L. *P < 0.05 and ***P < 0.001, by two-way ANOVA followed by Bonferroni's correction. Data are shown as the mean ± SEM in bar charts. The numbers in the flow cytometry plots indicate the proportions of gated cells.

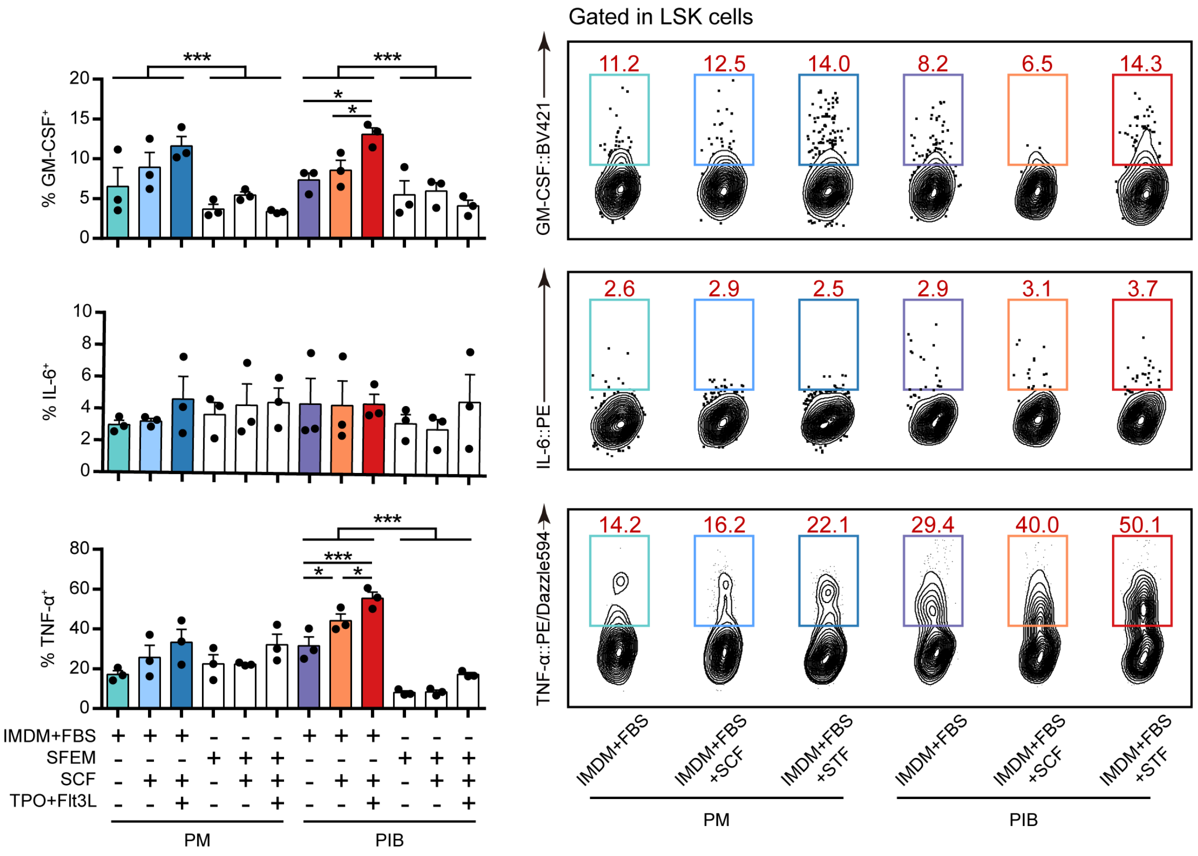

Supplemental Figure 5. Optimization of the culture system during the restimulation of *in vitro* Hepa SPSC-SN-induced HSPCs. The percentages of GM-CSF+, IL-6+, and TNF-α+ cells in LSK cells exposed to Hepa SPSC-SN were evaluated by flow cytometry. Naïve LSK cells were cultured in SFEM supplemented with 50 ng/ml SCF and 5% (v/v) Hepa mice-derived SPSC-SN for 4 days. After that, cells were washed and cultured in the indicated medium for an additional 6 h before staining. SCF, 50 ng/ml; TPO, 20 ng/ml; FLT3L, 100 ng/ml. PIB, the combination of PMA, ION, and BFA; PM, PMA plus MN. STF, SCF + TPO + Flt3L. *$P < 0.05$ and ***$P < 0.001$, by two-way ANOVA followed by Bonferroni's correction. Data are shown as the mean ± SEM in bar charts. The numbers in the flow cytometry plots indicate the proportions of gated cells.

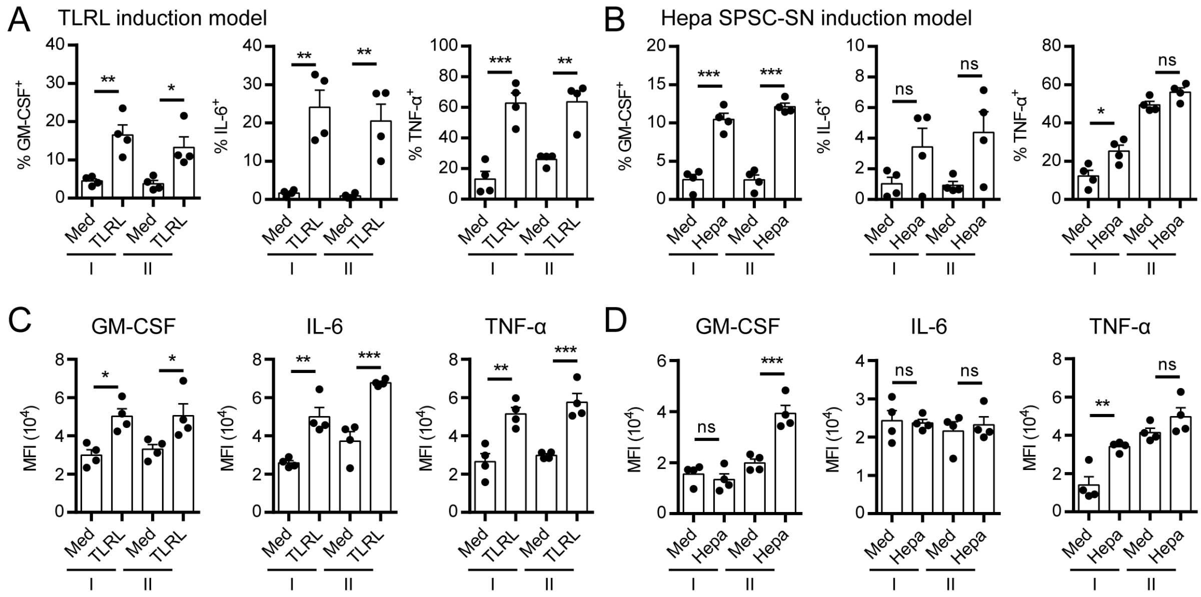

Supplemental Figure 6. The effect of restimulation I and restimulation II. (**A** to **D**) Naïve LSK cells were cultured in the indicated medium before restimulation. After that, cells were washed and cultured in the restimulation conditions as indicated for an additional 6 h before staining. (**A** and **B**) The percentages of GM-CSF$^+$, IL-6$^+$, and TNF-α$^+$ cells in LSK cells exposed to LPS and Pam3CSK4 (**A**) or Hepa SPSC-SN (**B**). (**C** and **D**) The median fluorescence intensity (MFI) of GM-CSF, IL-6, and TNF-α in the corresponding cytokine-positive cells. Data are shown as the mean ± SEM. Med, control medium (SFEM + SCF); TLRL, control medium supplemented with 100 ng/ml LPS and 1 μg/ml Pam3CSK4; Hepa, control medium supplemented with 5% (v/v) Hepa mice-derived SPSC-SN. *P < 0.05, **P < 0.01, ***P < 0.001; ns, not significant, by 2-way ANOVA followed by Tukey's test.

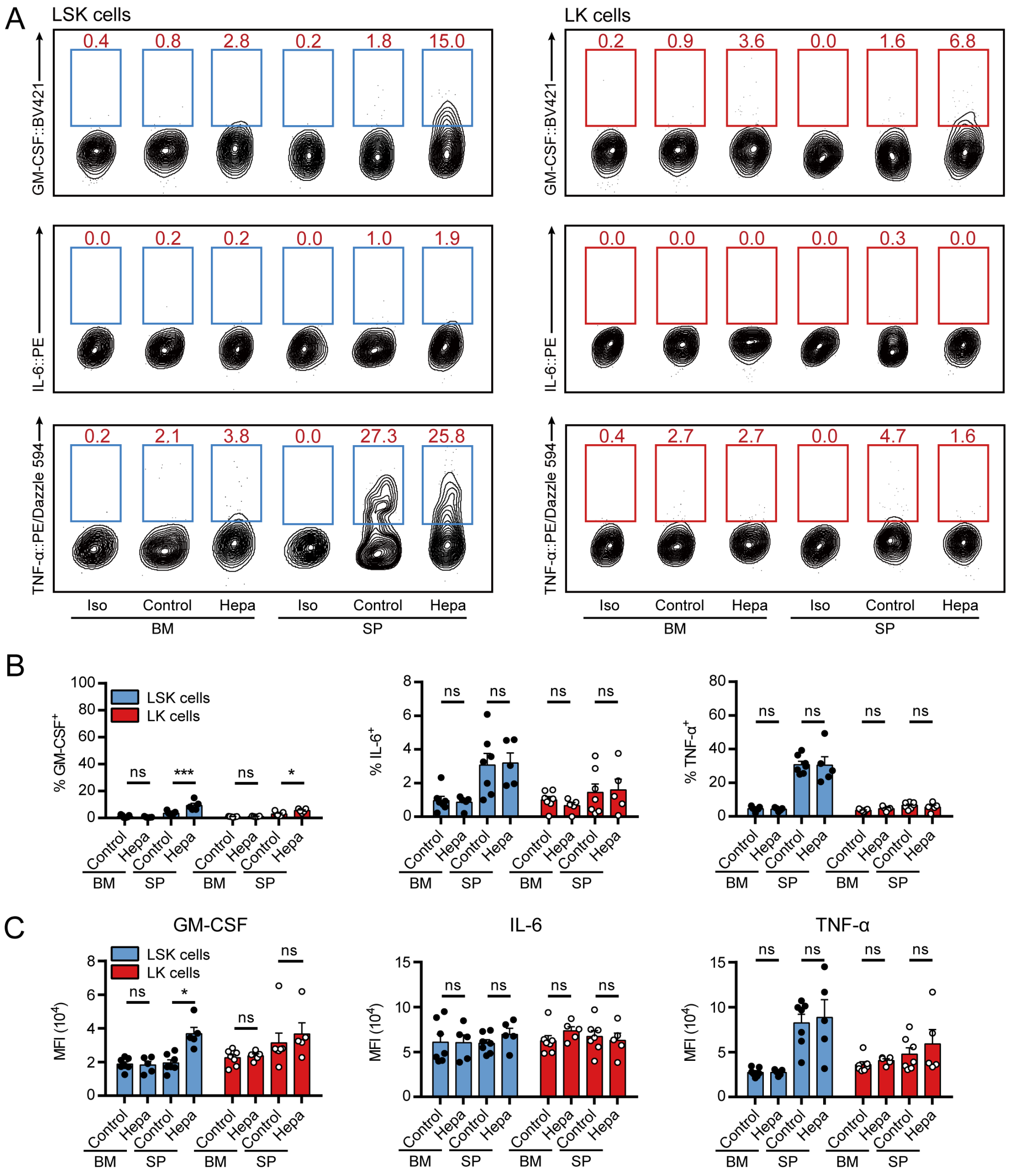

Supplemental Figure 7. Measuring intracellular cytokine levels in freshly isolated HSPCs from hepatoma-bearing mice with restimulation I. (**A**) Representative flow cytometric analysis of intracellular GM-CSF, IL-6 and TNF-α in freshly isolated LSK cells (left) and LK cells (right) from the BM and spleen of control or Hepa mice. The numbers in the flow cytometry plots indicate the proportions of gated cells. The data refer to a typical experiment out of three that generated similar results. Iso, isotype antibody control. (**B**) The percentages of GM-CSF$^+$, IL-6$^+$, and TNF-α$^+$ cells in LSK and LK cells (*n* = 5–7 per group). (**C**) The median fluorescence intensity (MFI) of GM-CSF, IL-6, and TNF-α in the corresponding cytokine-positive cells (*n* = 5–7 per group). Data are shown as the mean ± SEM. *P < 0.05, **P < 0.01, ***P < 0.001; ns, not significant, by 2-way ANOVA followed by Tukey's test.

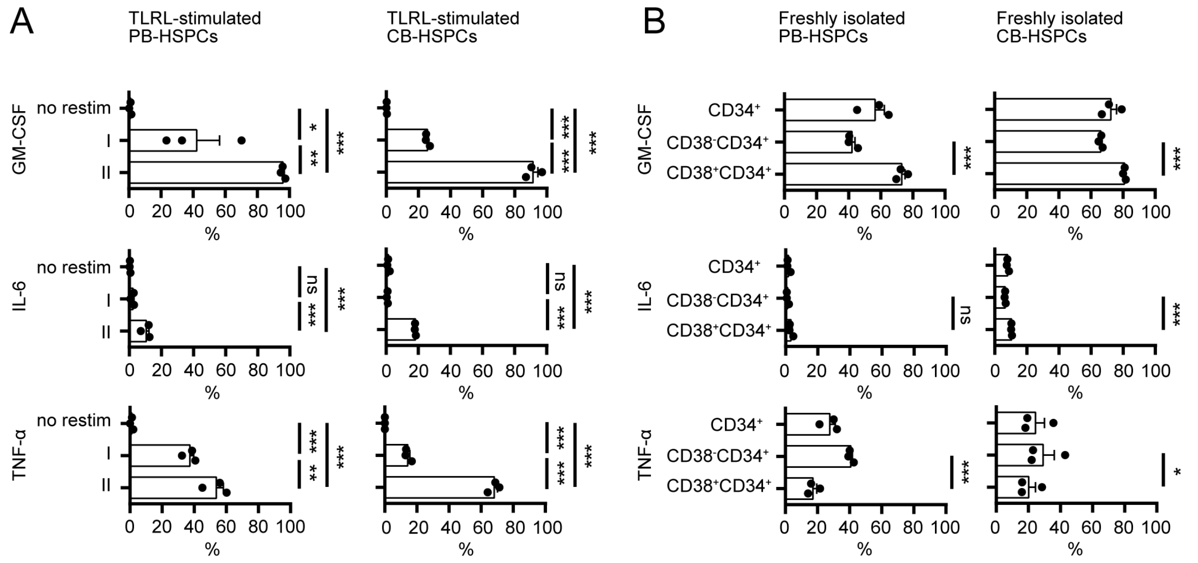

Supplemental Figure 8. Cytokine expression levels in stimulated and freshly isolated human HSPCs. (**A**) The percentages of GM-CSF$^+$, IL-6$^+$, and TNF-α$^+$ cells in TLRL-stimulated human blood-derived HSPCs (*n* = 3 per group). To obtain a sufficient number of HSPCs, lin$^-$CD34$^+$ cells were first expanded in SFEM supplemented with 50 ng/ml SCF. On day 3, these cells were exposed to LPS and Pam3CSK4 for 12 h, and then cells were harvested for flow cytometry analysis (with restimulation I). (**B**) The percentages of GM-CSF$^+$, IL-6$^+$, and TNF-α$^+$ cells in freshly isolated human blood-derived HSPCs (*n* = 3 per group). Cells isolated from fresh blood were treated with restimulation I before flow cytometric analysis. Data are shown as the mean ± SEM. *$P < 0.05$, **$P < 0.01$, ***$P < 0.001$; ns, not significant, by 1-way ANOVA followed by Tukey's test.

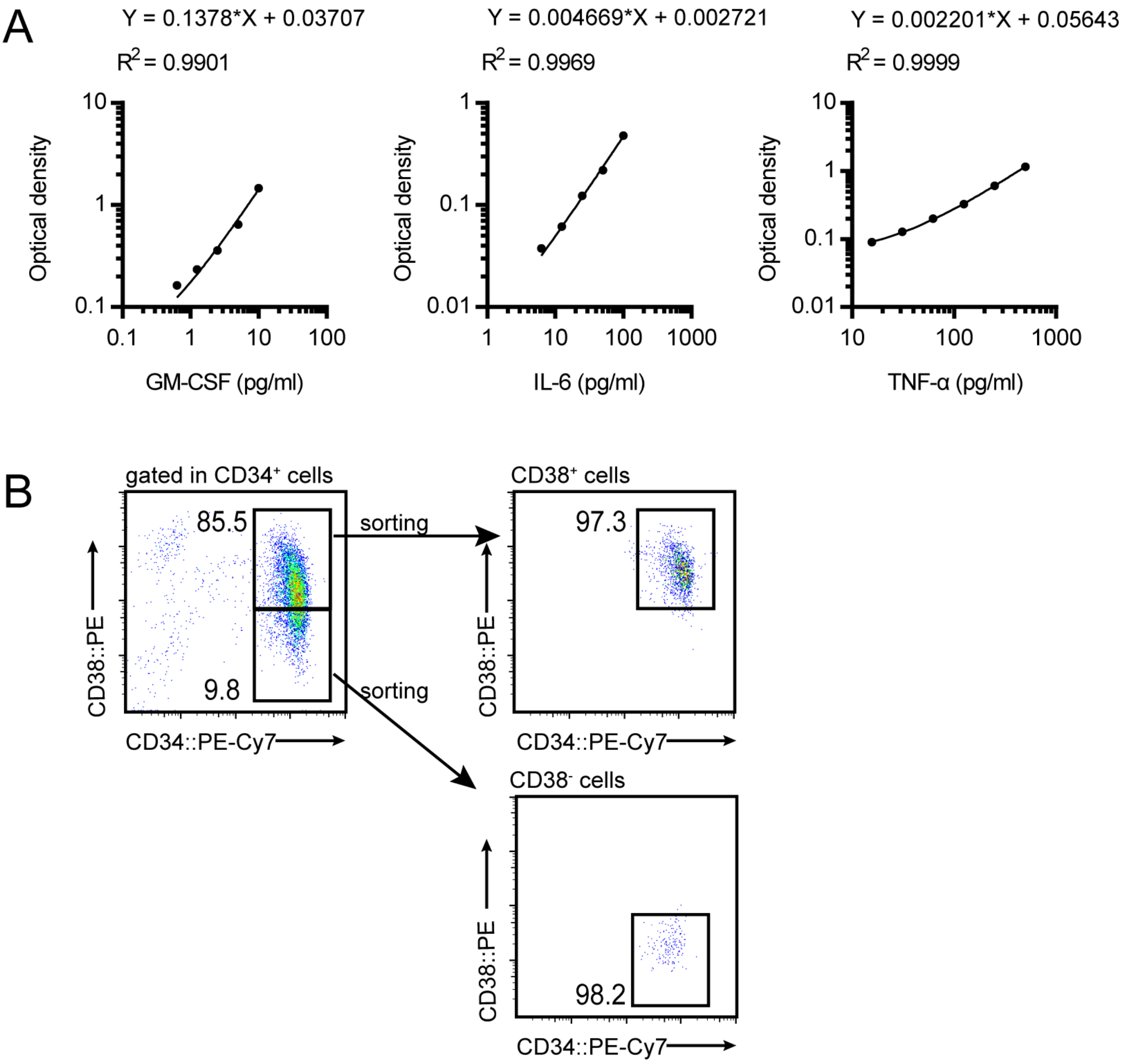

Supplemental Figure 9. Cytokine production and secretion by human blood-derived HSPCs. (**A**) ELISA standard curves for measuring human GM-CSF, IL-6, and TNF-α. (**B**) Representative flow cytometry plots showing the gating strategy and sorting purity of human $CD34^+CD38^-$ cells and $CD34^+CD38^-$ cells. The numbers in the flow cytometry plots indicate the proportions of gated cells.

Supplemental Table 1. Antibodies used for flow cytometry

Antigen	Reactivity	Fluorochrome	Clone	Supplier
**CD117**	Mouse	PC7	2B8	BD Biosciences
**CD117**	Mouse	Brilliant Violet 421	2B8	BioLegend
**CD11b**	Mouse	PE-Cy7	M1/70	BD Biosciences
**CD45**	Mouse	Brilliant Violet 570	30-F11	BioLegend
**cγRIII/FcγRII**	Mouse	-	2.4G2	BD Biosciences
**GM-CSF**	Mouse	Brilliant Violet 421	MP1-22E9	BD Biosciences
**Rat IgG2a, κ Isotype Control**	Mouse	Brilliant Violet 421	R35-95	BD Biosciences
**IL-6**	Mouse	PE	MP5-20F3	BD Biosciences
**Rat IgG1, κ Isotype Control**	Mouse	PE	R3-34	BD Biosciences
**Lineage Cocktail (CD3e, CD11b, CD45R/B220, Ly76, Ly6G, Ly6C)**	Mouse	PE-Cy5.5	145-2C11, M1/70, RA3-6B2, TER-119, RB6-8C5	BD Biosciences
**Lineage Cocktail (CD3e, CD11b, CD45R/B220, Ly76, Ly6G, Ly6C)**	Mouse	APC	145-2C11, M1/70, RA3-6B2, TER-119, RB6-8C5	BD Biosciences
**Sca-1**	Mouse	Alexa Fluor 700	D7	eBioscience
**Sca-1**	Mouse	FITC	D7	eBioscience
**TNF-α**	Mouse	PE/Dazzle 594	MP6-XT22	BioLegend
**Rat IgG1, κ Isotype Control**	Mouse	PE/Dazzle 594	RTK2071	BioLegend
**CD34**	Human	PE-Cy7	581	BioLegend
**CD38**	Human	PE	HIT2	BD Biosciences
**CD45**	Human	Krome Orange	J.33	Beckman Coulter
**GM-CSF**	Human	PE-CF 594	BVD2-21C11	BD Biosciences

**Rat IgG2a, κ Isotype Control**	Human	PE-CF594	R35-95	BD Biosciences
**IL-6**	Human	APC	MQ2-13A5	BioLegend
**Rat IgG1, κ Isotype Control**	Human	APC	RTK2071	BioLegend
**Lineage Cocktail (CD3, CD14, CD16, CD19, CD20, CD56)**	Human	FITC	UCHT1, HCD14, 3G8, HIB19, 2H7, HCD56	BioLegend
**TNF-α**	Human	eFluor 450	Mab11	eBioscience
**Mouse IgG1 κ Isotype Control**	Human	eFluor 450	P3.6.2.8.1	eBioscience

Abbreviations: APC, allophycocyanin; Cy, Cyanine; FITC, fluorescein isothiocyanate; PE, phycoerythrin.

Supplemental Table 2. List of reagents used in the study

Reagent	Supplier	Catalog No.	Final Conc.
**Anti-biotin Microbeads**	Miltenyi Biotec	130-090-485	-
**BFA**	BioLegend	420601	5 μg/ml
**CD117 MicroBeads**	Miltenyi Biotec	130-091-224	-
**CD34 MicroBeads**	Miltenyi Biotec	130-046-702	-
**Collagenase I**	Sigma-Aldrich	C9891	1 μg/ml
**Collagenase XI**	Sigma-Aldrich	C17657-100MG	30 μg/ml
**ConA**	Sigma-Aldrich	C5275-5MG	5 μg/ml
**DNase I**	Sigma-Aldrich	DN25-100MG	50 μg/ml
**FBS**	Gibco	A31608	-
**Fixation/ Permeabilization**	BD Biosciences	554714	-
**GM-CSF Antibody**	eBioscience	16-7331-85	5 mg/ml
**Human Interferon alpha 2**	Sino Biological	13833-HNAY	100 ng/ml
**Human Interferon beta**	Sino Biological	10704-HNAS	10 ng/ml
**Human Interferon-gamma**	Sino Biological	11725-HNAS	50 ng/ml
**Hyaluronidase**	Sigma-Aldrich	H3506-100MG	50 μg/ml
**IL-6 Antibody**	BioLegend	504506	1 mg/ml
**IMDM**	Gibco	12200-036	-
**ION**	Merck Millipore	407951	1 μg/ml
**LPS**	Sigma-Aldrich	L2630	100 ng/ml
**Monensin**	eBioscience	00-4505-51	2 μM
**Pam3CSK4**	Invivo Gene	tlrl-pms	1 mg/ml
**PMA**	Sigma-Aldrich	D8139	25 ng/ml
**Recombinant Human IL-6**	R&D Systems	206-IL-010	50 ng/ml
**Recombinant Human SCF**	Sino Biological	10451-H08B	50 ng/ml
**Recombinant Human TPO**	Sino Biological	10381-H08H	20 ng/ml
**Recombinant Murine Flt3L**	PeproTech Inc.PeproTech	250-31L	100 ng/ml
**Recombinant Murine SCF**	PeproTech Inc.PeproTech	250-03	50 ng/ml
**Recombinant Murine TPO**	PeproTech Inc.PeproTech	315-14	20 ng/ml
**Red Blood Cell Lysis Buffer**	TBD Science	NH4CL2009	-
**RPMI 1640**	Thermo Fisher Scientific	C11875500BT	-
**SFEM**	STEMCELL TechnologiesSTEMCELL	09650	-

**TNF-α Antibody**	eBioscience	16-7321-81	5 mg/ml

Abbreviations: SFEM, Serum-free medium; IMDM, Iscove' s modified Dulbecco's medium; FBS, fetal bovine serum; SCF, Stem Cell Factor; TPO, Thrombopoietin; Flt3-Ligand, Fms-related tyrosine kinase 3 ligand; LPS, lipopolysaccharide; Pam3CSK4, Pam3CysSerLys4; PMA, phorbol 12-myristate 13-acetate; ION, ionomycin; BFA, brefeldin A; ConA, concanavalin A; RPMI 1640, Roswell Park Memorial Institute 1640 Medium.
